# The Vitality of Epithelial Cells and the Etiology of Cancer

**Published:** 1899-04

**Authors:** 


### The Vitality of Epithelial Cells and the Etiology of Cancer.

What the nature of the irritant may be that causes the localized overgrowth of epithelial cells which we call cancer, we are yet no nearer knowing than we were before the demonstration of its exact pathology, more than half a century ago. Notwithstanding all the claims that have been made of the causal influence of external biologic factors, parasites from bacteria, and fungi, scizzomycetes, and blastomycetes to various forms of animal parasites, gregarines and protozoa generally, we are no nearer the solution of the problem than we were before.

Of late the subject has been approached from the other side, the essential vitality of epithelial cells and their reaction to various irritants, and some most interesting results have been obtained by various observers. In Dr. Hektoen's review of this subject for the first number of *"Progressive Medicine"** (the advance sheets of

*"Progressive Medicine", a Quarterly Digest of New Methods, Discoveries, and Improvements in the Medical and Surgical Sciences. Volume I, No. 1, March, 1899. Edited by Hobart A. Hare, M. D. Lea Brothers & Co., New York and Philadelphia.

which are in our hands), we find some striking observations on the subject collated. Ljunggren, a Scandinavian physician, for instance, found to his surprise that he could preserve carefully sterilized bits of human skin in sterile human ascitic fluid for months, and that the cells of the tissues retained their vitality. Three months after their removal from the body the cells of the deeper layers showed well stained nuclei, and good protoplasmic structure. Successful transplantation was made with pieces kept in such sterile fluid for a month. Small pieces of the transplanted skin were removed at varying intervals, and it was found that a marked proliferation of epithelial cells showing many nuclear figures had occurred. Special precautions were taken, which absolutely assured the absence of cells that might have grown in from the surrounding cutaneous margin and so vitiated the conclusions. The transplanted cells not only grew over the raw surface, but penetrated, also, into the granulation tissue beneath, after the manner of a beginning carcinomatous growth.

Almost more interesting and suggestive than this are the observations made by Loeb here in America on epithelial regeneration. The abstracts of them by Dr. Hektoen in *"Progressive Medicine"* is so clear and succinct that we copy part of it verbatim: "From the margin of a tissue-defect huge epithelial protoplasmic or plasmodial masses move in a sliding manner over the naked surface, inclosing and dissolving the crust and other obstacles. Regenerating epithelium readily removes such substances as cartilage when placed in its way. Below the protoplasmic layer epithelial cells wander in from the margins of the defect, and often grow down into the connective tissue, apparently checking the growth of the latter. The process is closely allied to changes in carcinoma. At the same time active changes, such as mitoses, occur in the epithelial cells removed some distance from the margins of the wound * * * Loeb believes that the wandering of the cells, as outlined, is in response to stereotropism, and forms a determining in inducing mitosis in the remaining cells." The pregnant significance of these observations, especially the apparent action at a distance of epithelial elements in arousing epithelial cells into reproductive and germinal activity, can scarcely be overestimated. This is the essence of carcinoma, though in healthy subjects the vital resistance may be sufficient to restrain the morbid overgrowth that would otherwise result.

According to Loeb, "if a small bit of epithelium is placed in the centre of the crust covering a defect in the skin, it begins to send out processes in all directions into the crust, the cells acting as separate organisms, independent of blood supply or nervous influence." We are evidently closely in touch, in these manifestations, with the as yet inexplicable vital forces that we see at work in all their untrammelled energy and power in cancer. Further observations are needed to give the deductions from these observations practical application. They constitute, however, the most hopeful aspect of the present pathological work on cancer as far as regards the near prospect of discovering its etiology. Their value as additions to biological science, especially to that mysterious problem, the struggle for life among the various cells of the body tissues, can scarcely be overestimated.

